# P-542. Characteristics and Outcomes of Parainfluenza Virus Infection Among Patients with Sickle Cell Disease

**DOI:** 10.1093/ofid/ofaf695.757

**Published:** 2026-01-11

**Authors:** Sapna Pardasani, Jose Amadeo A Ferrolino, Ronald H Dallas, Megan Peterson, Pamela Merritt, Amanda Cole, Amber Davis, Ashleigh Gowen, Tina Culley, Kim J Allison, Randall Hayden, Parul Rai, Diego R Hijano

**Affiliations:** St. Jude Children's Research Hospital, Dallas, TX; St. Jude Children's Research Hospital, Dallas, TX; St. Jude Children's Research Hospital, Dallas, TX; St. Jude Children's Research Hospital, Dallas, TX; St. Jude Children's Research Hospital, Dallas, TX; St. Jude Children's Research Hospital, Dallas, TX; St. Jude Children's Research Hospital, Dallas, TX; St. Jude Children's Research Hospital, Dallas, TX; St Jude Children's Research Hospital, Memphis, Tennessee; St. Jude Children's Research Hospital, Dallas, TX; St. Jude Children's Research Hospital, Dallas, TX; St Jude Children's Research Hospital, Memphis, Tennessee; St. Jude Children's Research Hospital, Dallas, TX

## Abstract

**Background:**

Respiratory viral infections are a common cause of acute chest syndrome (ACS) in children with Sickle Cell Disease (SCD). Few studies have explored the role of parainfluenza virus (PIV) in this population. Hence, we aimed to describe the characteristics and clinical outcomes of PIV infection in patients with SCD.

**Figure:**
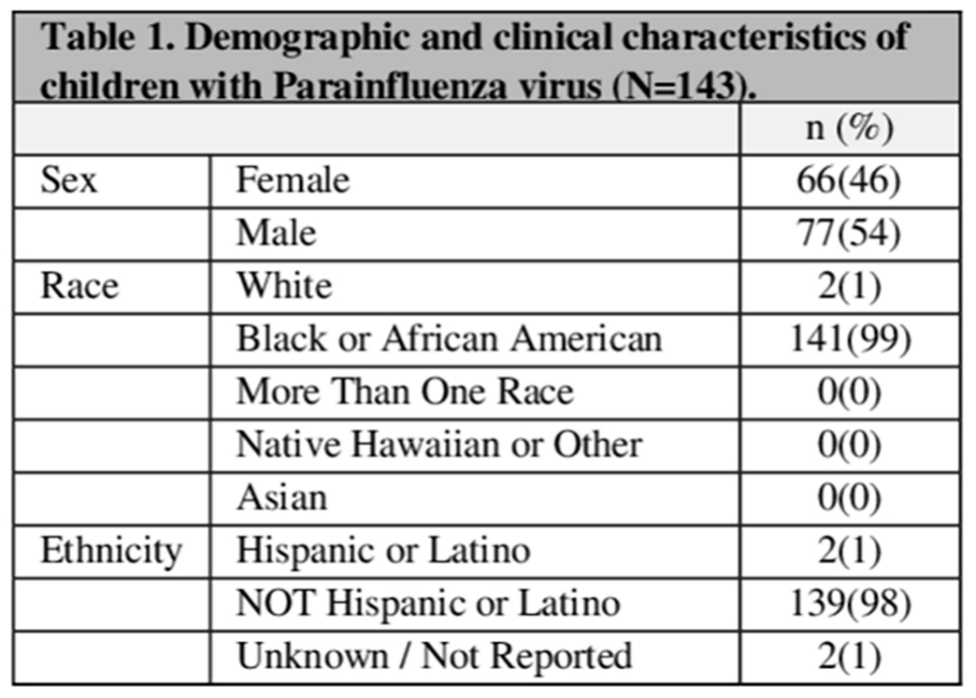

**Figure:**
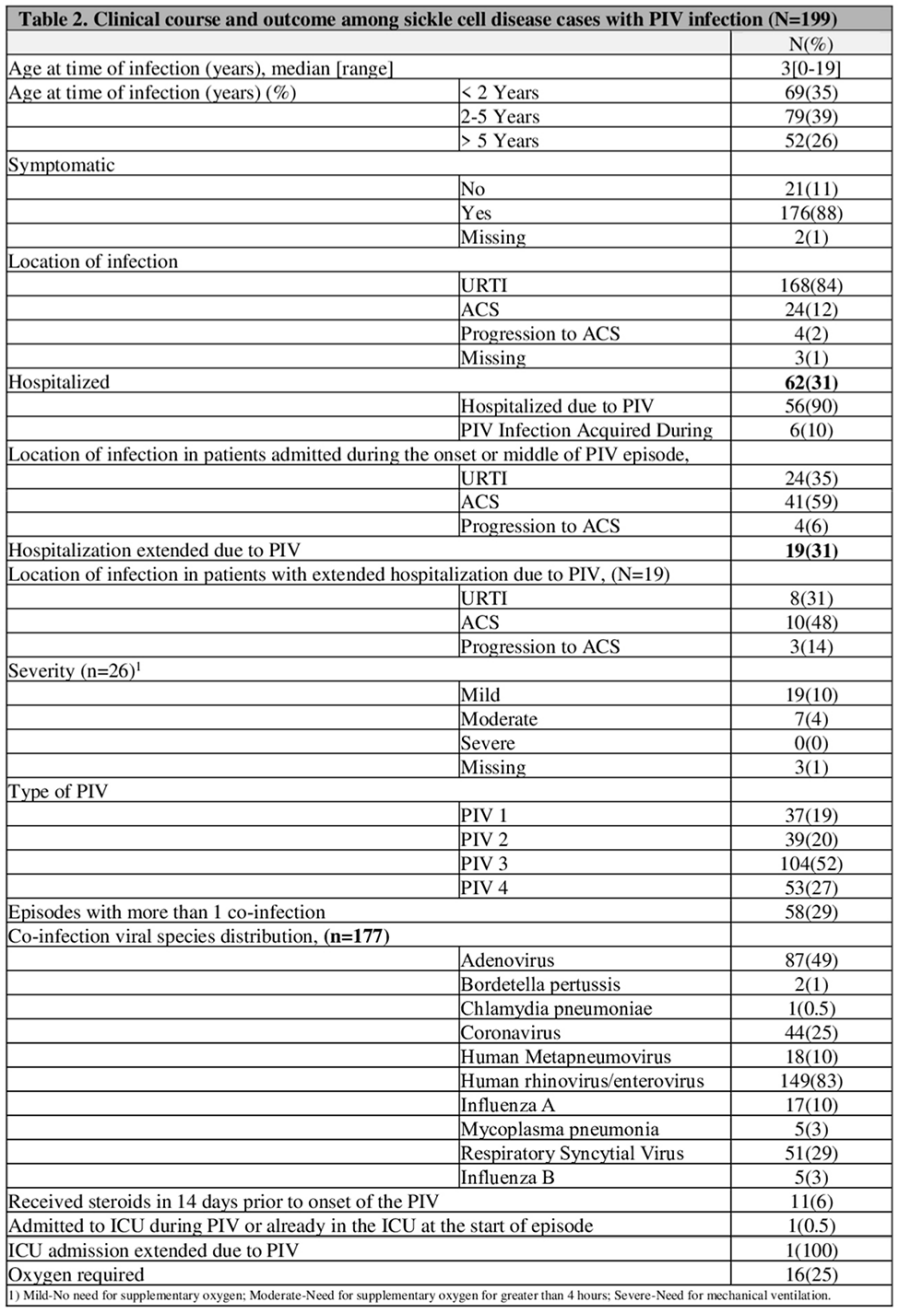

**Methods:**

This single-center study was conducted at St. Jude Children’s Research Hospital, included SCD children aged 21 years or younger who tested positive for PIV between 2014 and 2024. Data on demographics, clinical characteristics, treatment, and clinical outcomes were collected from electronic medical records.

**Figure:**
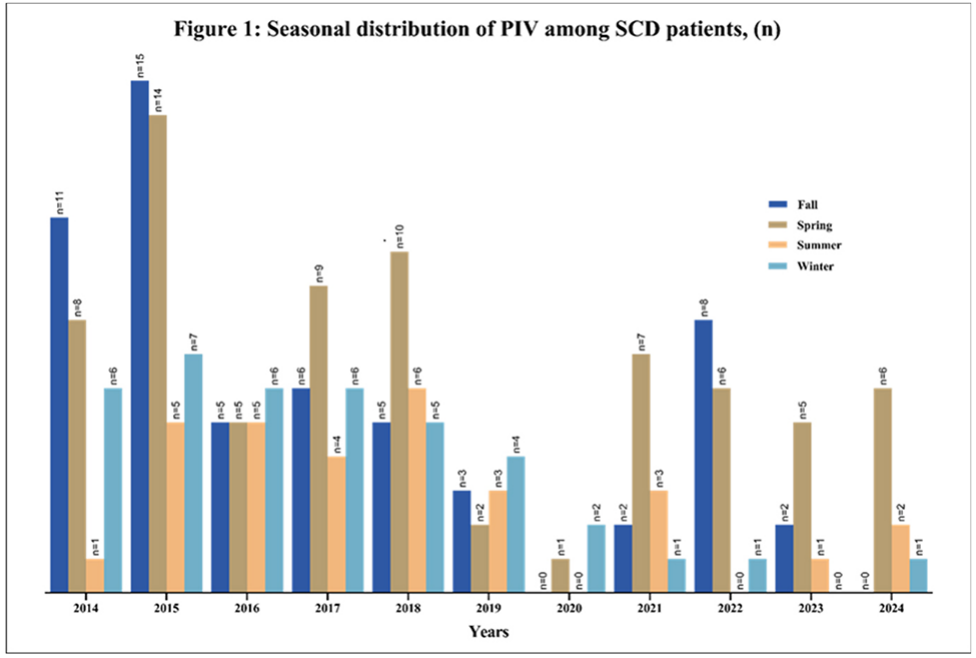

**Figure:**
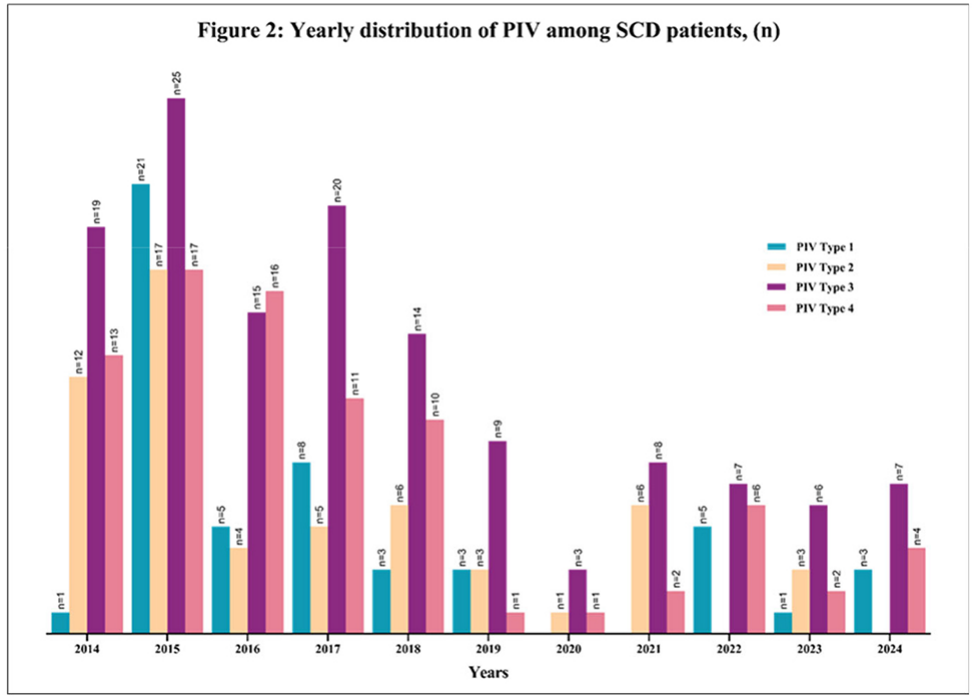

**Results:**

A total of 143 patients with SCD were included in the study, with 199 episodes of PIV documented. The cohort (n=143) was predominantly Black and non-Hispanic (98%) (Table 1). The median age was 3 years (range, 0-19). The most common PIV was PIV3 (52%), followed by PIV4 (27%), PIV2 (20%) and PIV1(19%). 29% of the episodes had more than one respiratory viral infection. Most PIV episodes (86%) presented with upper respiratory tract infection (URTI), and approximately 12% developed ACS. More than quarter of the episodes (n=56) required hospitalization due to PIV; one-third of these patients experienced extended hospitalization due to PIV (n=19), and a quarter (n=16) needed oxygen. One episode required admission to the intensive care unit (ICU) (Table 2).

**Conclusion:**

The findings highlight that PIV infections primarily manifest as upper respiratory tract infections, with some progressing to ACS. While most cases were managed without hospitalization, many required inpatient care, with some requiring extended hospital stays and oxygen support. Further research is needed to develop targeted interventions and optimize care strategies for SCD patients with PIV infections, potentially reducing complications and improving outcomes.

**Disclosures:**

Randall Hayden, MD, Abbott: Board Member|Abbott: Serving on the advisory board|Cepheid: Board Member|Cepheid: Serving on the advisory board|Roche Diagnostics: Advisor/Consultant|Roche Diagnostics: Board Member|Roche Diagnostics: Serving on the advisory board

